# Image based three-dimensional kinematic mapping to optimize femoral tunnel placement for intra-articular cranial cruciate ligament reconstruction in dogs

**DOI:** 10.3389/fvets.2026.1898326

**Published:** 2026-08-26

**Authors:** Caroline Robine-Decourcelle, Pierre-Yves Rohan, Christophe Muth-Seng, Sylvain Persohn, Mathieu Manassero, Véronique Viateau

**Affiliations:** 1 Ecole Nationale Vétérinaire d’Alfort, Université Paris-Est, Maisons-Alfort, France; 2 Arts et Métiers Institute of Technology, IBHGC – Institut de Biomécanique Humaine Georges Charpark, Université Sorbonne Paris Nord, Paris, France

**Keywords:** 3D kinematic, canine stifle joint, cranial cruciate ligament rupture, isometry, surgical planning, synthetic ligament

## Abstract

**Objective:**

The development of synthetic ligaments (SLs) has sparked renewed interest in intra-articular cranial cruciate ligament (CrCL) reconstruction. In this procedure, minimizing changes in graft length through optimal tunnel placement is essential for long-term function. However, three-dimensional (3D) assessment of isometry across the femoral attachment area in dogs remains limited, which prevents evidence-based optimization of tunnel positioning. Thus, the aim of this study was to perform strain-constrained, animal-specific mapping of the femoral region for intra-articular CrCL reconstruction in dogs and to identify drill-compatible sites for SL fixation that minimize strain during joint flexion–extension.

**Methods:**

Six pelvic limbs from large-breed dogs (mean body weight 27.7 ± 9.7 kg) underwent three-dimensional (3D) segmentation of bones and CrCL footprints using computed tomography (CT). Femorotibial kinematics during passive flexion–extension were computed using biplanar radiography and optical tracking. The ligament trajectories were modeled as straight segments connecting candidate mesh femoral nodes on the medial aspect of the lateral condyle to tibial nodes included within a 5 mm diameter circle centered on the centroid of the native CrCL footprint. Changes in trajectory length during a flexion–extension cycle, and the extent of contiguous femoral regions below selected strain thresholds were recorded.

**Results:**

Flexion–extension produced coupled triplanar stifle motions within the reported ranges. The femoral nodes with the lowest maximum strain clustered cranial and proximal to the centroid of the native CrCL footprint. A continuous 5 mm diameter region was consistently identified at a maximal strain threshold between 6.5–9%. Caudal and cranio-distal nodes relative to the native CrCL footprints exhibited the highest strain values (>30%).

**Conclusion:**

Subject-specific 3D kinematic modeling consistently identified drill-compatible femoral and tibial regions with a 9% maximal strain cut-off. Future refinement of the 3D kinematic model integrating active loading, soft-tissue mechanics, and SL architecture is required to support clinical translation.

**Clinical relevance:**

Positioning the femoral tunnel slightly cranially and proximally to the native CrCL footprint, and centering the tibial tunnel on its centroid, may improve SL longevity and long-term joint stability.

## 1 Introduction

Rupture of the cranial cruciate ligament (CrCL) is one of the most common causes of hindlimb lameness in dogs (1). Treatment of this condition is necessarily surgical (2), and the currently available reference treatments (Tibial Plateau Leveling Osteotomy, Tibial Tuberosity Advancement, Lateral Extracapsular Stabilization), despite satisfactory clinical results, do not restore normal joint biomechanics. Progression of degenerative joint disease and increased stiffness have been reported in the long term (3).

Intra-articular replacement of the ruptured ligament, analogous to anterior cruciate ligament (ACL) reconstruction in humans, offers the potential to re-establish more physiological joint mechanics. However, autograft or allograft options remain limited in dogs because of the lack of tendinous autografts of sufficient strength that can be harvested in this species without major functional impairment (4), and safety concerns with tendon allografts which require specific, cumbersome procedures (5). Synthetic ligaments (SLs) therefore represent an appealing alternative, yet their use has been hindered by high rupture rates up to 80% (6–10), limiting their widespread use in clinical settings. Among the identified causes of rupture, poor intra-articular positioning of the SL, particularly on the femoral tunnel site, has been identified in both humans and animals as a major contributor to failure, underscoring the importance of precise tunnel positioning during reconstruction (11–14). While the femoral tunnel allows for considerable flexibility in positioning, the tibial tunnel offers only little margin for adjustment. Its placement must respect surrounding structures, including the load-bearing menisci and intermeniscal ligaments, and therefore closely matches the native ligament’s footprint.

Anatomical placement of femoral and tibial tunnels within the ACL footprints is widely recommended in human to replicate the joint’s native biomechanics (15, 16). However, since the native ACL is not strictly isometric during motion (17, 18) and SLs tolerate length variation poorly (19), near-isometric reconstruction has been proposed (16, 20) to limit the strains exerted on the ligament and its fixation sites (21). Near-isometric reconstruction in people is defined as either a maximum lengthening of 1–3 mm (16, 19, 20) or falling within the 95% confidence interval of the native ACL elongation values (22). Given the wide anatomical variability of the canine stifle across breeds, there is no universally accepted displacement threshold that defines near-isometry in this species.

Even though anatomical placement of tunnels apertures within the native ACL footprints has been recommended in people (6–8, 10), there is currently no consensus on their optimal placement in dogs. It has been established that, as in humans where incorrect implantations account for 22% of graft failures (14), graft malposition leading to lack of isometry, excessive graft loading leading to plastic deformation or anchorage failure, are key causes of rupture in the animals model (13, 23). Optimal implantation parameters were also found to be animal-dependent (13), highlighting the need for a more individualized, precise, and predictable surgery that considers the animal specific anatomy and biomechanics to optimize their placement and fixation and ultimately restore joint stability.

In both human and animal research, subject-specific computed tomography (CT) based computational models combining fluoroscopy, EOS imaging, or motion capture data have enabled precise three-dimensional (3D) reconstruction of joint kinematics throughout the flexion–extension cycle (24–27). In parallel, separate studies have characterized CrCL footprints (28) or identified isometric points in the canine stifle (29). However, to our knowledge, no study has delineated isometric regions relative to native ligament insertion sites within subject-specific kinematic numerical models—information that is essential for clinically relevant SL placement, as ligament footprints constitute key anatomical landmarks for achieving isometric or near-isometric graft positioning in humans. Computational models in human ACL reconstruction (30) further illustrate the importance of subject-specific models to assess tunnel location, graft tension, and load distribution—yet such computational approaches remain unexplored in canine stifles.

The aim of this study was to identify a continuous femoral region on the medial aspect of the lateral femoral condyle that minimizes CrCL length variation during flexion–extension, and provides sufficient space for a 5 mm bone tunnel. To address this question, we developed a subject-specific 3D kinematic model based on CT and Polaris motion capture data in which the SLs were modeled as straight-line trajectories connecting their tibial and femoral attachment sites.

We hypothesized that a specific region on the medial aspect of the lateral femoral condyle could be identified that would be compatible with current surgical drilling standards in dogs, while also maintaining the minimum possible strain over a physiological motion range.

## 2 Materials and methods

### 2.1 Anatomic specimens

The experiments were conducted on six pelvic limbs collected from six large-breeds dogs (20–40 kg) without age or sex restrictions that were euthanized at our institution for reasons unrelated to the study and whose owners donated the body between September and October 2022. Animals were selected to represent the three main morphological types (dolichomorphic, mesomorphic, and brachymorphic). The sample included three shepherd dogs, one American Staffordshire terrier, one Labrador retriever, one shepherd crossbreed, and one German shorthaired pointer. None of the dogs had a reported history of osteoarticular disease. Direct and indirect drawer tests (tibial compression test) performed on the explanted limbs showed no evidence of cranial tibial translation, consistent with a stable stifle. Kinematic measurements of cranial tibial translation performed during an other part of the experimental protocol (data not shown) and subsequent anatomical dissection of the specimens further confirmed the absence of stifle instability and the macroscopic integrity of the CrCL in all cases. The limbs were labeled sequentially by increasing body weight (PA_01–PA_06). One specimen was notably cachectic prior to euthanasia (13.5 kg). This specimen was nonetheless retained for analysis to preserve interbreed representativeness.

All experimental procedures complied with European regulations governing animal experimentation (86/609/CEE, 1986; 2010/63/UE, 2010). All pelvic limbs were explanted by coxofemoral disarticulation and subsequently frozen at −20 °C. The limbs were freeze-thawed once for the completion of CT-scans (to construct the 3D computer model) and a second time for the kinematic testing. Thawing was performed at 4 °C for 48 h prior to each.

### 2.2 Construction of a generic model from CT and regionalization

Following preparation, each limb was scanned using a CANON AQUILION lightning SP 81 CT scanner (Canon Medical System Corp, Otawara, Japan) with the stifle joint positioned at an angulation corresponding to a physiological standing position (135°). The acquisition parameters were: 120 kV; mA modulation: “quality °” dose regulation; rotation time: 0.5 s; field of view: 280 mm; focus: small; collimation: 0.5 × 40; “detail” pitch: 0.625. Two images’ reconstructions were generated: a soft tissue reconstruction using a standard abdomen filter (slice thickness 1 mm, increment 0.8 mm), and a 30US2 bone filter (slice thickness 0.5 mm, increment 0.3 mm).

Bone segmentation (femur, tibia, patella, fibula, fabella, and sesamoid bones) was performed using the semi-automatic graph cut module (31) of the Medical Imaging Interaction Toolkit (MITK-GEM_2017.5.0; German Cancer Research Center—Division of Medical Image Computing, Heidelberg, Germany). The graph cut algorithm was initialized using user-defined foreground and background labels. The resulting segmentations were visually inspected and manually refined as needed to ensure accurate bone contours. All segmentations were performed by a single operator. The intraobserver reproducibility of the segmentation procedure was assessed by computing the mean and standard deviation of the point-to-surface distance across three repeated segmentations of limbs from three specimens by the same operator.

The surface meshes resulting from the segmentation were decimated and the number of triangles were reduced from approximately 150,000 to around 30,000 using Geomagic 21.0 software (Geomagic U.S. Corp., Research Triangle Park, NC, USA). This reduction facilitated subsequent image processing and produced a more uniform tessellation of the surface geometry. To enable a direct comparison of anatomical locations across specimens, the surface mesh of the median specimen was deformed to match each individual femur’s anatomy while maintaining point-to-point correspondence between homologous anatomical regions. This process ensured that equivalent surface locations could be identified consistently across all specimens, despite differences in bone morphology.

### 2.3 Flexion–extension kinematic testing

Each limb was dissected to preserve only the joint structures including the joint capsule, cruciate ligaments, lateral and medial menisci, the collateral ligaments and the distal portion of the quadriceps muscle, the patella, and the patellar tendon, thereby maintaining the integrity of the stifle joint continuity and the extensor apparatus. All tests were carried out at room temperature, with the specimens regularly moistened using physiological saline to preserve tissue hydration. The experimental setups were adapted from previously developed test benches used for the mechanical evaluations of SL implantation in ewes (13, 32, 33).

**Figure 1:**
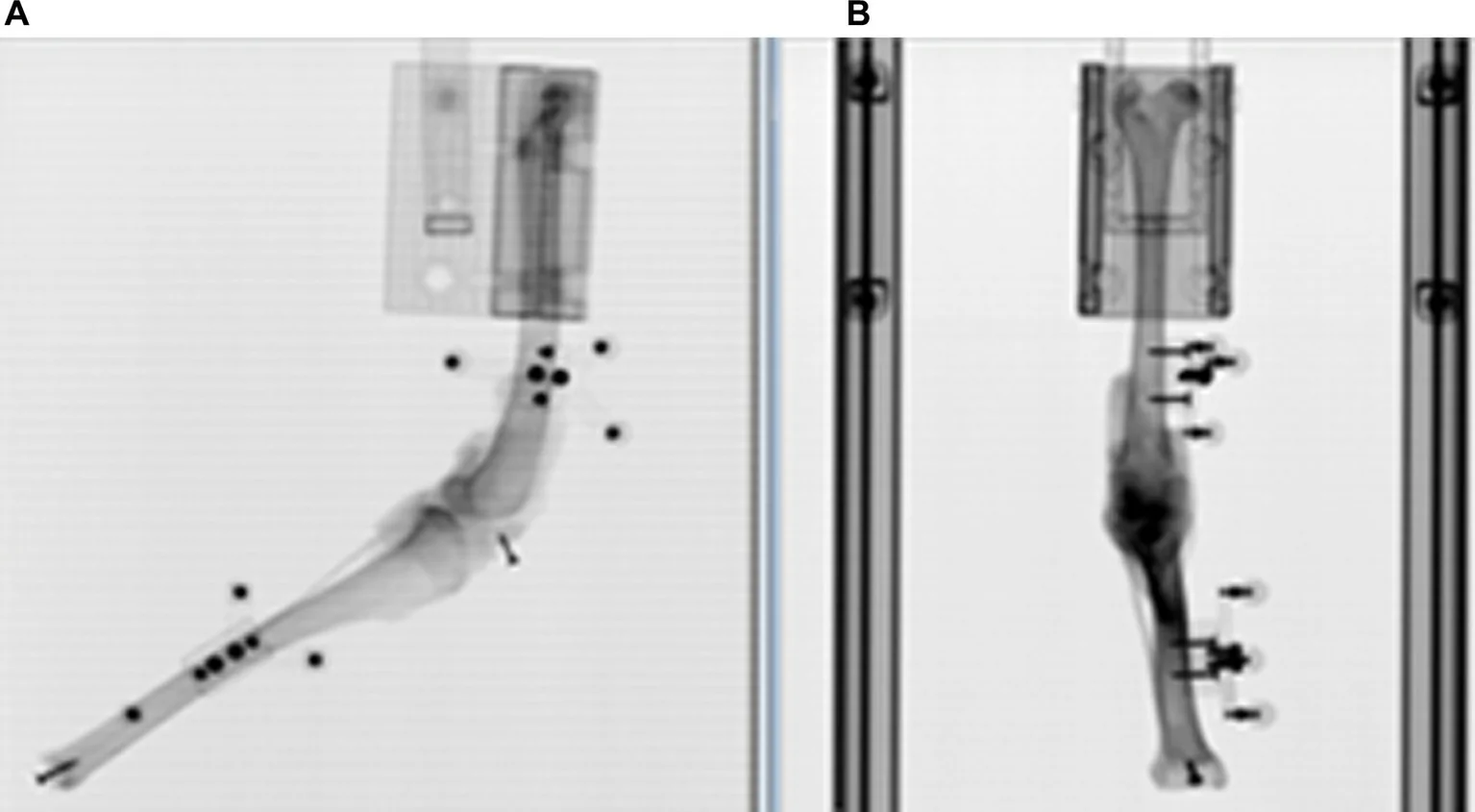
Limb mounted in the EOS system with tripods of infrared markers screwed to the femur and the tibia, profile **(A)** and frontal **(B)** views.

Three-marker rigid clusters (tripods) equipped with passive infrared reflective markers were rigidly attached to the femoral and tibial bone segments using screws and tracked using a 3D optoelectronic motion capture system (Polaris, NDI, Waterloo, Ontario, Canada), which uses two integrated infrared cameras to determine the spatial position of passive markers within a calibrated measurement volume. Biplanar radiographs of both bones were acquires with an EOS imaging system (EOS, EOS Imaging, Paris, France; 90 kV, 200 mA) (Figure 1). The 3D surface mesh of the femur and tibia were then reconstructed using a previously described method (34) and already adapted for animal use with sheep (31). Marker positions on each tripod were simultaneously detected, and their 3D coordinates were obtained in the EOS system of reference.

The femur was rigidly fixed, and the motor was connected to the quadriceps tendon using screws, thread guides, and a force sensor. To ensure reproducible specimen positioning and preserve the physiological line of action of the quadriceps, custom-made clamps were designed from the CT-based reconstructions and 3D printed. To maintain physiological patellar tracking, the traction cable was aligned with the patellar tendon, and a guide positioned around the patellar screw maintained the patella centered within the trochlear groove throughout the motion. The force sensor continuously monitored the tensile load transmitted through the quadriceps tendon. An initial 20 N quadriceps force was applied to provide a standardized and reproducible tension of the extensor mechanism during passive flexion–extension. The objective was to reproduce the unloaded passive range-of-motion assessment performed intraoperatively in humans during intra-articular ACL reconstruction, where flexion–extension movements are used to evaluate graft isometry and detect potential conflicts or impingement.

**Figure 2:**
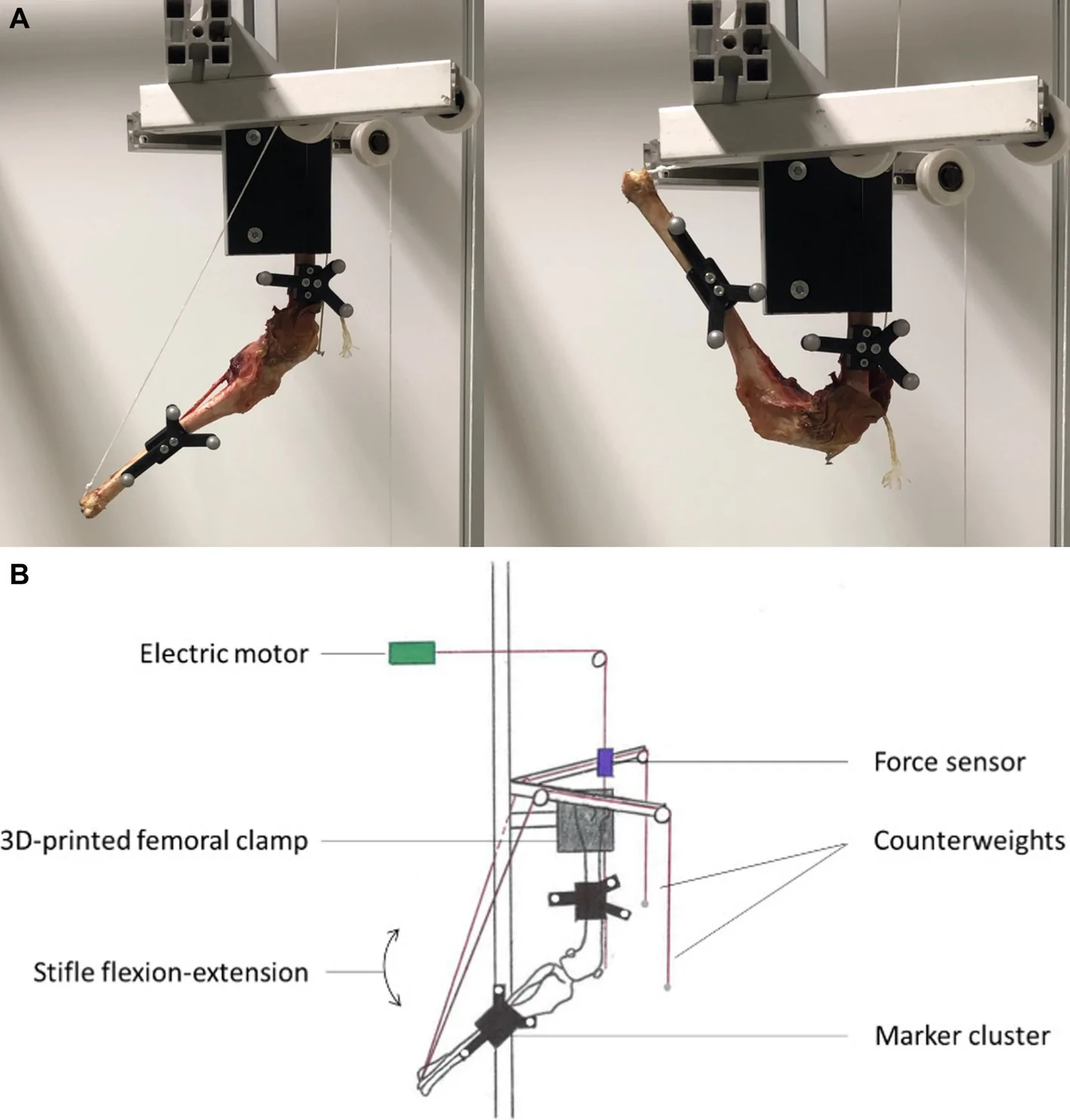
Experimental setup for ex vivo kinematic testing. **(A)** Photograph of the custom mechanical testing apparatus showing the initial position (left), and maximum flexion (right). **(B)** Schematic representation of the experimental setup as viewed from the optical tracking system.

Flexion–extension was performed under angular position control. A restoring force was applied to the distal tibia through a malleolar screw connected to a pulley system and two 100 g counterweights, which maintained the distal tibial cables under tension throughout the motion while generating negligible restoring force in the initial position. The custom clamping system constrained the femur while allowing free tibial varus–valgus and internal–external rotations. Each specimen underwent six continuous load-unload cycles that were conducted at a rate of 0.015°/sec, ranging from the initial position to at least 90° of flexion (Figure 2).

**Figure 3:**
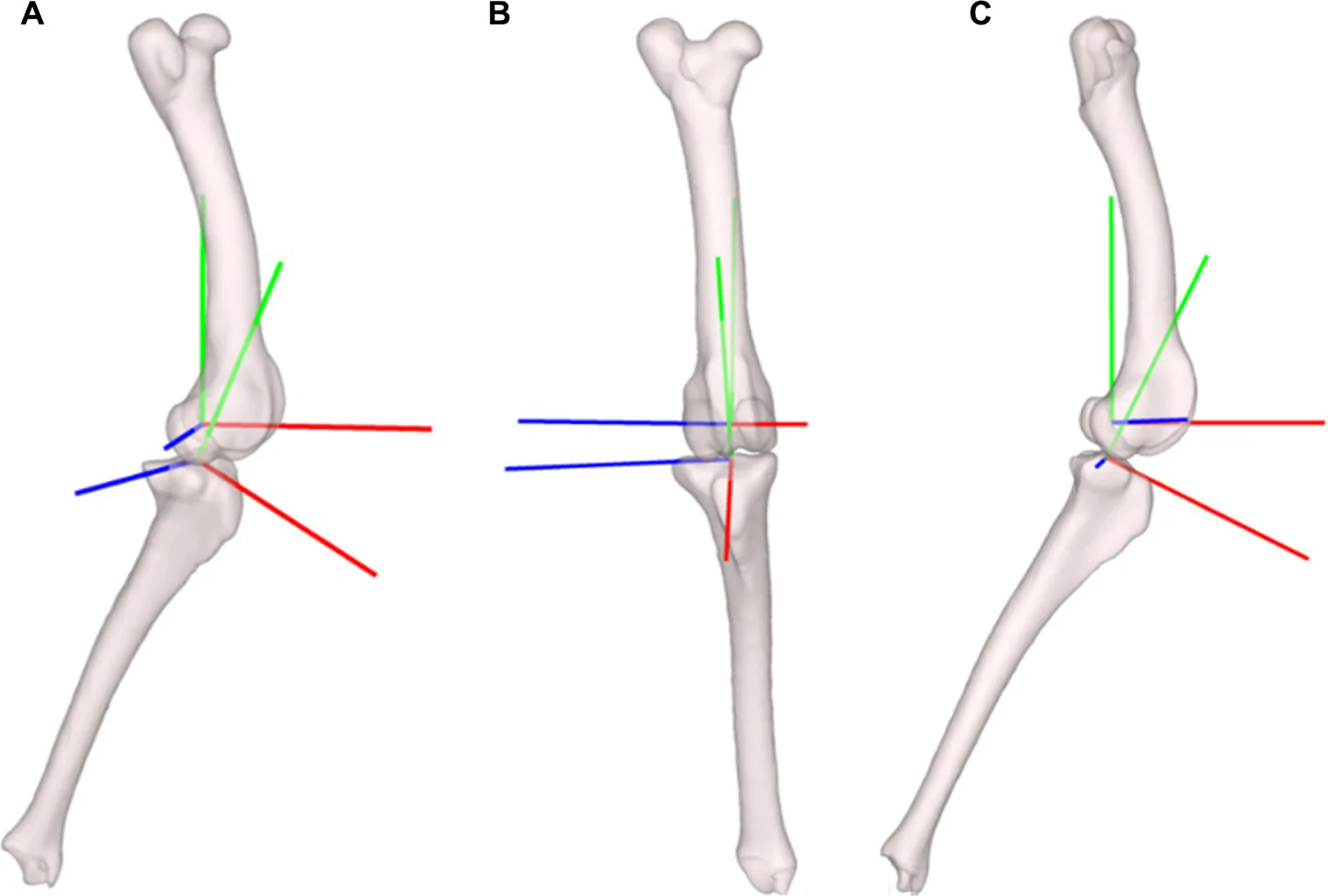
Anatomical frames for each bone: profile **(A),** frontal **(B),** and oblique **(C)** views with the frontal (*Rx*, red), sagittal (*Rz*, blue), and transverse (*Ry*, green) axes.

Anatomical coordinate systems were defined in accordance with the work of Schlatterer et al. (35). For each 3D femoral and tibial model, an orthonormal reference frame was established from anatomical landmarks identified on the corresponding femoral and tibial surface meshes, respectively. The frames were constructed by analogy with previously published approaches in animals (36, 37) and with methods validated in humans (34, 38) (Figure 3). The identified anatomical landmarks were used to define the origin and orientation of the coordinate axes for each bone.

The biplanar radiographs enabled computation of the transform matrices between the anatomical frames of bone structures and the corresponding technical tripod frames following a previously published method (24, 26, 32). Since the tripods were tracked at 20 Hz (volumetric accuracy: 0.25 mm; 95% CI: 0.5 mm), the motion of the anatomical reference frame could be accurately determined throughout flexion–extension testing. During data acquisition, the tracking camera was positioned approximately 1.5 m from the tripods, with no obstacle situated between the reflective markers and the Polaris, to ensure that all reflective marker arrays remained within the measurement volume throughout the flexion–extension cycle.

The variation of relative position of the tibia with respect to the femur was calculated based on mobile axis, with the following sequence (center of rotations): *zy*′*x*″ (*y*′ denoting the mobile *y* axis after the first rotation about *z*, and *x*″ denoting the mobile *x* axis after the first 2 rotations). The three components of translation as well as the internal–external rotation and varus–valgus of the tibia related to femur were computed as a function of the flexion angle. The final cycle was retained for analysis to minimize the influence of transient viscoelastic effects and tissue settling. To allow interspecimen comparison, the initial flexion angle was standardized across all tests.

The experimentally recorded flexion–extension motion was then reproduced on the three-dimensional femoral and tibial meshes by applying the rigid transformations obtained from the optical tracking system. For each position of the flexion–extension cycle, the corresponding mesh coordinates were used to calculate ligament trajectory length variations. All calculations were performed in MATLAB (MathWorks, Natick, MA, USA).

Following biomechanical testing, the joint capsule was removed, and the CrCL was carefully excised, preserving its femoral and tibial attachment areas (footprints). These footprints, corresponding to the bone-ligament interface regions, were marked using a 50:50 preparation of varnish mixed with barium sulphate (MICROPAQUE®) to enhance their radiopacity. A subsequent CT enabled precise identification of the marked footprints. The resulting segmentation data were integrated into the existing 3D bone models, allowing accurate localization of the ligament attachment areas. Finally, the surface meshes of the CrCL footprints were projected onto the complete bone meshes, generating an enhanced 3D reconstruction and animation that visualized the CrCL insertion sites throughout motion.

### 2.4 Quantitative mapping of CrCL isometry during flexion–extension cycle from multiple candidate points

**Figure 4:**
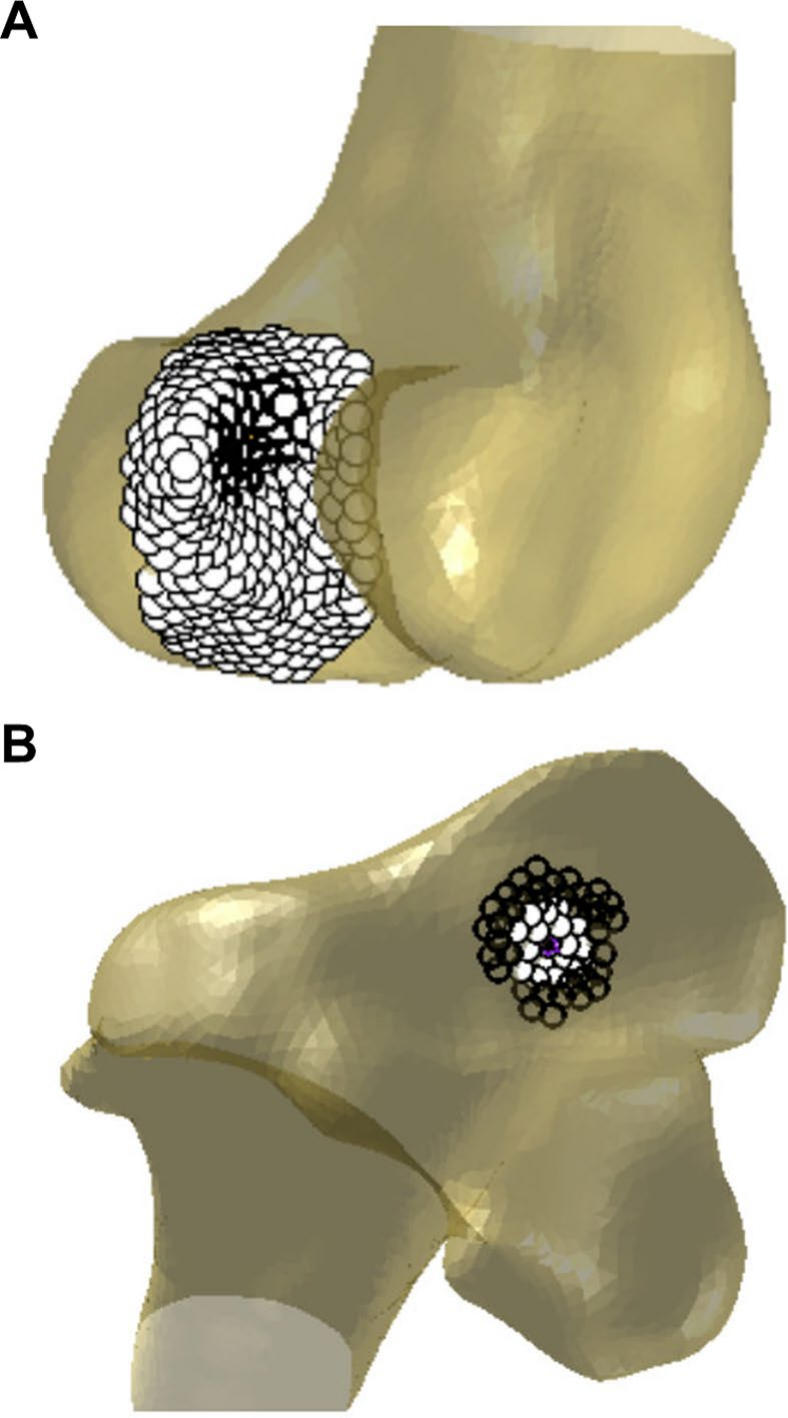
Regions of interest selected on the femoral and tibial surface meshes. **(A)** Medial aspect of the lateral femoral condyle. Mesh nodes selected for the analysis are shown as white circles outlined in black. These included nodes located within the native femoral CrCL footprint which are indicated by a thicker black outline. **(B)** Tibial region of interest. Mesh nodes selected for the analysis are shown as white circles. This included mesh nodes located within the native tibial CrCL footprint which are indicated by a thick black outline. The centroid of the native CrCL footprint is represented by a black star.

The SL was modeled as a straight line connecting one femoral point and one tibial point. The centroid of each native tibial and femoral CrCL footprint was determined by computing the area-weighted average of all vertices of the respective surface meshes. The mesh node closest to this centroid was then designated as the centroid of the footprint. Mesh nodes belonging to the medial aspect of the lateral femoral condyle were manually identified using an in-house software tool for interactive selection of regions of interest on surface meshes while preserving the original mesh topology and node numbering. Three hundred mesh nodes were selected for the analysis. The selected nodes on the tibial side comprised all the nodes located within the tibial footprint (Figure 4).

On the tibial side, the nodes of interest were defined as those located within a distance equal to the reference drilling radius (i.e., 2.5 mm) from the centroid of the native CrCL footprint. The reference drilling diameter was set at 5 mm, consistent with the drilling diameter of bone tunnels most commonly used in dogs for intra-articular CrCL reconstruction.

The initial distance *L*0 between each femoral-tibial node pair was defined as the distance between these points at a joint angle of 135° of extension, corresponding to the physiological standing position of large breeds of dogs (39). For each position of the flexion–extension cycle, the internodal distance was computed, and the corresponding strain was expressed as:

$$\varepsilon =\frac{L-L0}{L0}$$

For each candidate femoral node, strain was first calculated for every trajectory connecting this node to each tibial node located within the reference drilling radius throughout the flexion–extension cycle. For each trajectory, the maximum strain over the entire cycle was retained. The highest of these maximum strain values was then assigned to the corresponding femoral node. This conservative approach ensured that the selected femoral location satisfied the strain criterion for all potential ligament trajectories within the planned tibial tunnel.

To visualize the spatial distribution of these values, a color-coded strain map of the medial aspect of the lateral femoral condyle was generated for each limb, with each femoral mesh node coloured according to its assigned maximum strain value.

**Figure 5:**
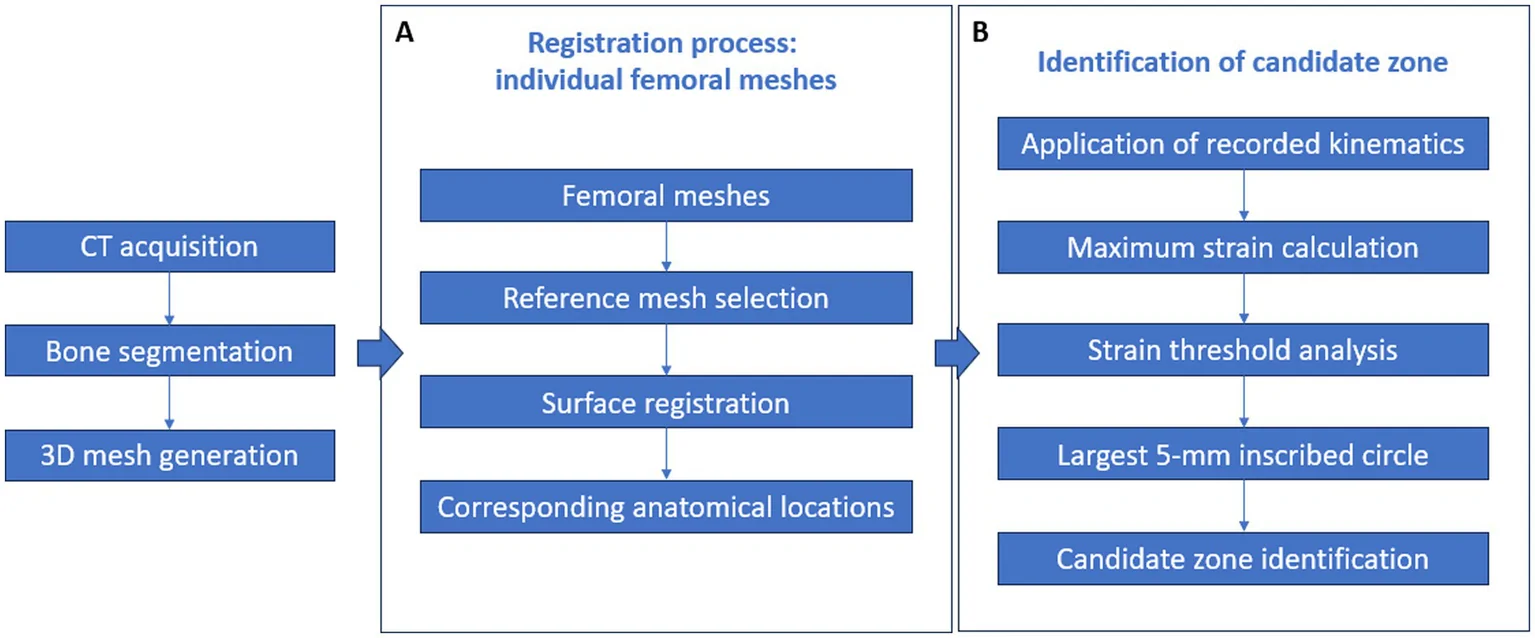
Schematic representation of the workflow used to identify the candidate isometric zone. **(A)** Individual femoral meshes were registered to establish anatomical correspondence between specimens. **(B)** Maximum strain values were then calculated for each femoral location during joint motion, and the region fulfilling predefined strain criteria while allowing a 5 mm inscribed circle was identified.

Finally, the femoral area exhibiting the smallest maximum strain while still allowing an inscribed circle of at least 5 mm—matching the drilling diameter—was identified (candidate zone). For each predefined strain threshold (2–15%, in 0.005% increments), the femoral mesh nodes with maximum strain equal to or less than the selected threshold were identified. Then, the diameter of the largest possible inscribed circle within this region was calculated iteratively. The main steps of the computational workflow used to identify the candidate femoral zone are summarized in Figure 5.

## 3 Results

### 3.1 Anatomic specimens

The mean dog age was 9.3 ± 6.0 years (median: 8.7 years, range: 0.7–16.2) and the mean body weight was 27.7 ± 9.7 kg (median: 26 kg, range: 13.5–39.2).

### 3.2 Biomechanical testing

**Figure 6:**
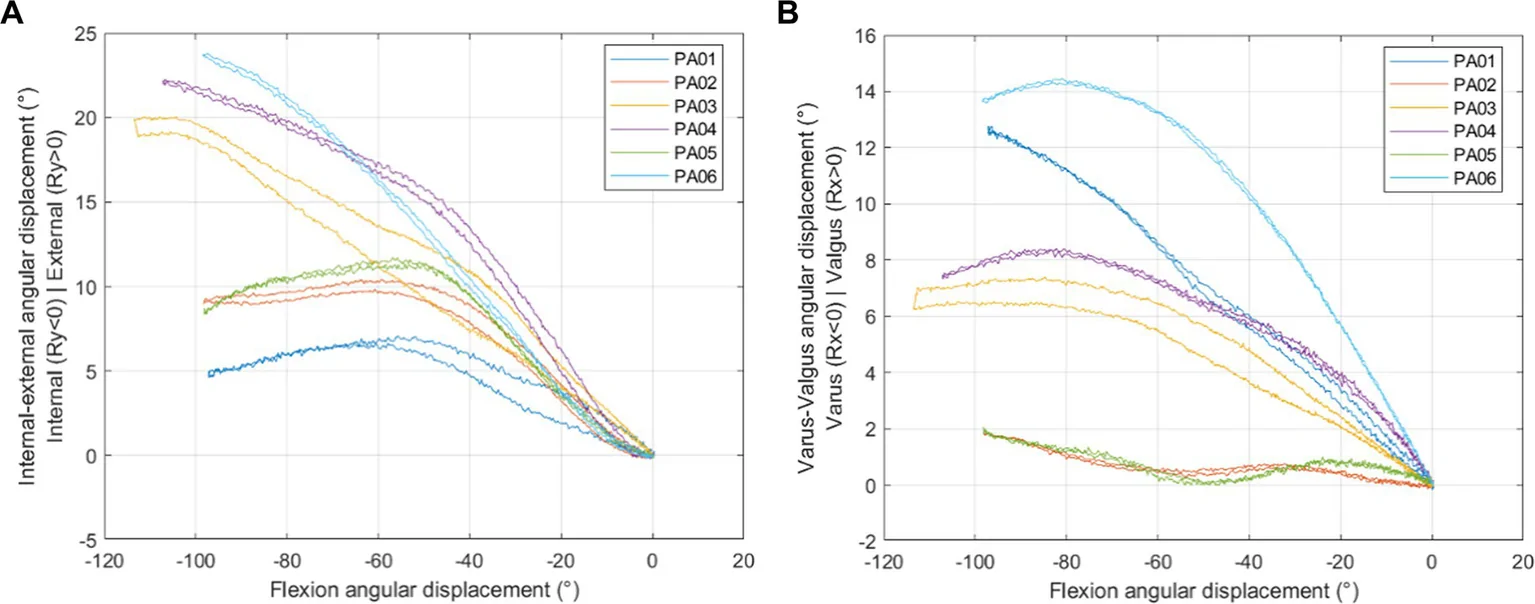
**(A)** Internal–external rotation and **(B)** Varus–valgus rotation of the tibia relative to the femur as a function of flexion–extension angle. Each color represents one of the six canine specimens.

For each specimen, two displacement curves in varus–valgus (Rx) and in internal–external rotation (Ry) were plotted as a function of the flexion–extension angle (Rz), as illustrated in Figure 6. The overall range of motion during testing extended from 155.7 ± 8.2° in flexion to 53.3 ± 7.9° in extension.

The test results showed considerable interindividual variability in both the amplitude and pattern of angular displacement during flexion–extension. This variability was observed for both internal–external rotation and varus–valgus rotation. The amplitudes of motion ranged from 1.8° to 14.4° in varus–valgus (median: 7.8°) and 6.5 to 23.8° in internal–external rotation (median: 15.8°).

### 3.3 Uncertainty of the semi-automatic segmentation

Evaluation of the intraobserver reproducibility of the manual CT-based segmentation showed a high degree of consistency across repeated trials. The mean overlay error ranged from 0.001 to 0.26 mm for the femur and from 0.007 to 0.36 mm for the tibia, considering both segmentation excesses and defects. These values confirm that segmentation uncertainty remained well below the spatial resolution of the CT images, ensuring negligible impact on subsequent geometric analyses.

### 3.4 Femoral colormaps and variation in the diameter of the candidate zone as a function of the strain threshold

**Figure 7:**
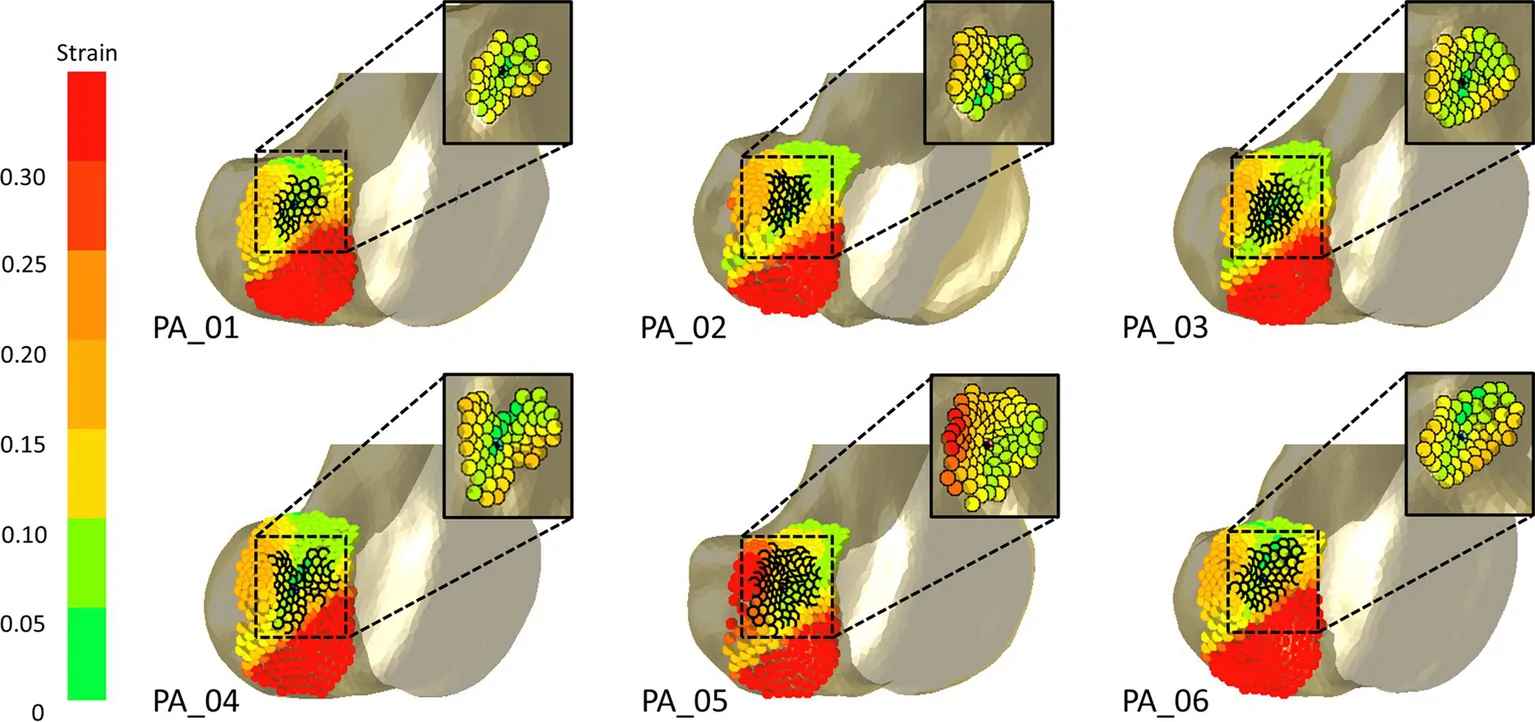
Spatial distribution of maximum strain across candidate femoral attachment sites. Color-coded maps represent the maximum strain calculated between each candidate femoral point located on the medial aspect of the lateral femoral condyle and the corresponding tibial nodes of interest throughout the complete flexion–extension cycle. Lower strain values (green) indicate regions where ligament length variations are minimized (greater isometry), whereas higher strain values (red) indicate greater variations in ligament length. For each specimen (PA_01–PA_06), the centroid of the native CrCL footprint (black-circled points) is represented by a black star, with a zoom in on the footprint.

For the tibial nodes positioned within 2.5 mm (the reference drilling radius) of the centroid of the CrCL footprint, the regions of the femoral condyle comprising the nodes with the least amount of strain throughout the flexion–extension cycle were located cranially and proximally to the centroid of the femoral footprint, as shown in Figure 7. Conversely, caudal or distal regions of the femoral condyle resulted in greater changes in trajectories length throughout the motion cycle.

**Figure 8:**
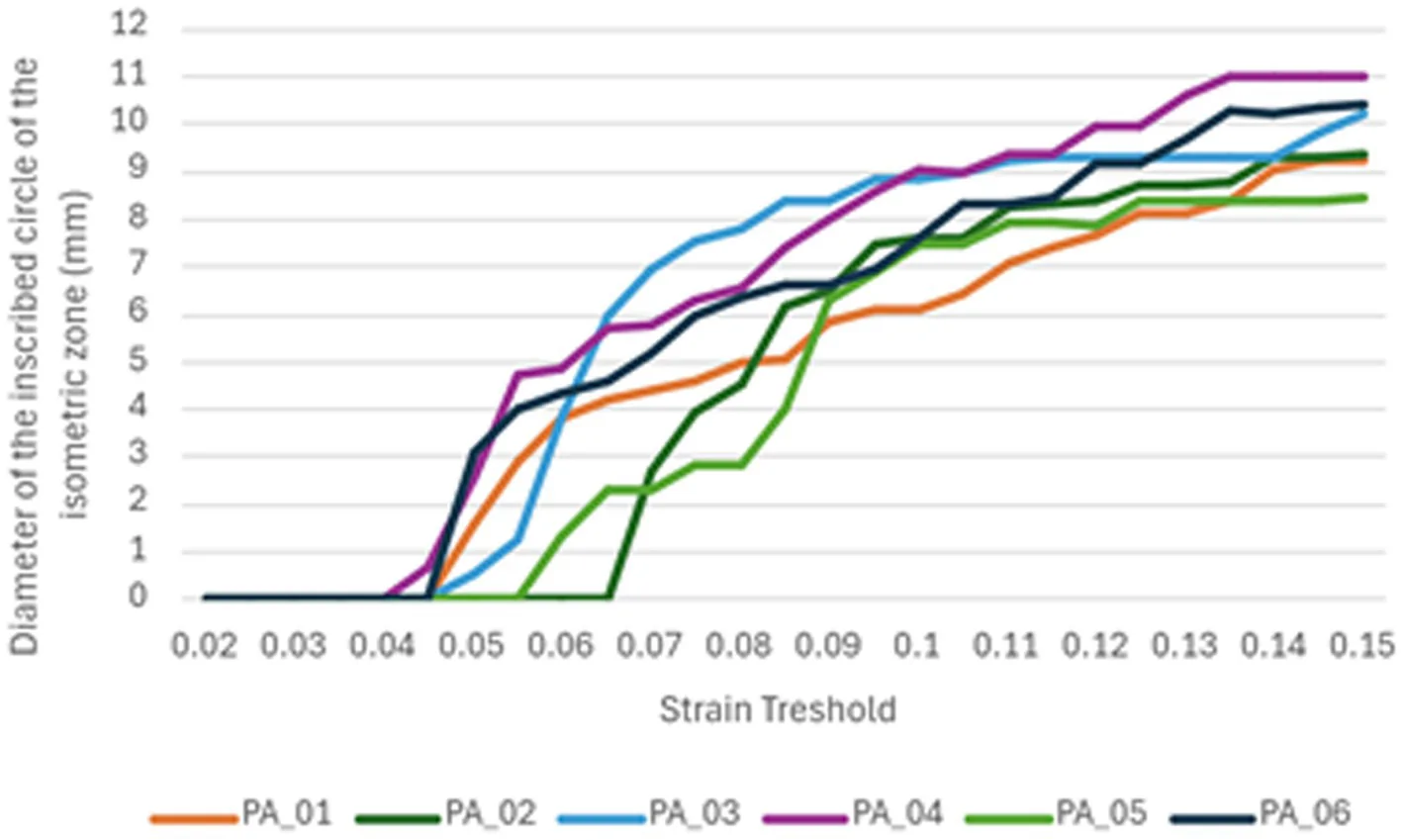
Quantitative assessment of isometric zone size according to strain threshold. The diameter of the largest inscribed circle within the region meeting each predefined strain threshold is plotted as a function of the allowable strain variation. This analysis quantifies the reduction in the size of the isometric region as increasingly restrictive strain criteria are applied, providing a summary measure of the extent of the most isometric femoral attachment area across specimens.

Despite morphological diversity and interindividual kinematic variability, all specimens exhibited an area with a diameter of at least 5 mm, located cranio-proximally to the centroid of the native CrCL footprint, and comprising nodes with strain thresholds between 6.5 and 9% (Figure 8). The femoral tunnel can be positioned within this zone to minimize the variation in the length of the trajectories throughout flexion–extension for each specimen. However, for a same diameter, the allowable strain variation significantly differed between animals, in some cases nearly doubling.

All specimens showed heterogeneous maximum deformation distribution among the nodes within the native CrCL footprint. In all specimens, some nodes within the CrCL footprint exhibited maximum deformations of over 15%. Specimen PA_05, whose native CrCL footprint extended more caudally than the others, exhibited nodes in the caudal region of its footprint with peak deformation reaching over 30%.

## 4 Discussion

The aim of this study was to determine whether, for a region centered on the centroid of the native tibial CrCL footprint, a femoral region on the medial surface of the lateral condyle could maintain near-constant intra-articular trajectories length throughout flexion–extension, and be compatible with standard 5 mm drilling. By combining identification of CrCL footprints with functional assessment using a subject-specific 3D kinematic numerical model derived from CT-based anatomy and experimentally measured motion data, we were able to quantify variations of trajectories lengths across the full flexion–extension cycle and identify femoral and tibial regions of minimal strain variation. The strength of this work lies in its integration of detailed anatomical analysis, precise mapping of the CrCL footprint location, and a functional investigation of CrCL isometry in dogs—a method that is not yet documented in the literature.

This study provides, to our knowledge, the first functional, strain-constrained 3D mapping of isometry along femoral and tibial anchor-point-defined trajectories for CrCL ligament reconstruction. Unlike the anatomical approach by Lin et al. (28), which estimated CrCL footprints using deformable bone templates, our method combines anatomical segmentation with motion-based modeling. This enabled assessment of length variation directly throughout the range of motion.

Yair et al. (29) proposed a preliminary 3D model of isometric points in a single dog, and reported isometric points caudally to the femoral footprint of the CrCL, with a length variation cut-off of 0.2 mm during the flexion–extension cycle. However, the motion cycle in the latter study ranged from only 131 to 80° of flexion and it has been demonstrated in humans that ligament length variation depends on the angle, and that the more the knee is extended, the less the ACL is isometric (17, 18). Furthermore, their study lacked anatomical registration of the dog’s CrCL footprints. The current study builds on this work by expanding the analysis to six limbs with individualized morphologies and a broader range of motion.

Although the passive flexion–extension configuration does not reproduce *in vivo* loading conditions, it mirrors the intraoperative conditions under which surgeons assess isometry during ligament implantation. The kinematic amplitudes recorded were consistent with published values for both ex vivo (40, 41) and in vivo (27, 42) tests, confirming that the bench setup reproduced physiological motion with satisfactory accuracy. The submillimetric segmentation precision (≤0.36 mm) and the consistent strain patterns across specimens support the robustness and repeatability of the proposed computational approach. The sample size (*n* = 6) was selected to capture interindividual variability in stifle morphology and kinematics among breeds predisposed to spontaneous CrCL rupture, rather than to enable statistical inference. This exploratory design prioritized anatomical and functional diversity over numerical power, allowing for a robust qualitative assessment of isometric behavior across morphotypes. The interindividual kinematic variability observed further supports the need for personalized, image-based surgical planning to optimize SLs positioning.

The strain and stiffness of SLs are dependent strongly on their composition and architecture and differ markedly from those of the native ligament (43, 44). In addition, these parameters can vary when cyclic loads are applied, modifying the initial biomechanical properties. Some studies have shown that fatigue cyclic tests can increase SL elongation (43), leading possibly to a recurrence of laxity while other studies have shown that low loads can improve mechanical properties by reorienting the fibers and act as a pre-conditioning (45). Variations of SLs properties over time illustrate the importance of isometric placement to limit the fatigue effect when the SL is exposed to cyclic forces, tolerance to an isometric defect being dependent on the nature of the implanted SL.

SLs are designed to mimic the nonlinear and viscoelastic behavior of native ligaments. The stress–strain curves of SLs do not clearly delineate the elastic and plastic regions, and biomechanical results are highly dependent on strain-rate, with damage beginning progressively. In the absence of a well-defined elastic limit for SLs used in dogs, it was not possible to define a critical threshold beyond which plastic deformation of these ligaments would occur. Instead of providing such a cut-off, we proposed a colormap that highlights zones on the medial aspect of the lateral femoral condyle that undergo the greatest and least strain during the flexion–extension cycle.

In humans, it is well established that the ACL is not fully isometric during the range of motion (17, 18). Also, some authors have shown that the most isometric zone on the femur is close to the center of the ACL insertion (46), while others have shown that the center of the footprint is less isometric than anteromedial insertion sites (47). It was also suggested that the most isometric setting would be achieved by either an ‘over-the-top’ positioning of the femoral tunnel directed anteroinferiorly or by a drill hole positioned at the anterosuperior corner of the femoral footprint (48). The posterior zone of the ACL footprint has also been identified as the least isometric (49). Our findings were consistent with those of these studies. We found that the nodes exhibiting the least amount of strain throughout the flexion–extension cycle were located cranially and proximally to the centroid of the femoral CrCL footprint, and that the caudal or distal regions of the femoral condyle resulted in greater amounts of strain. Although there is a general trend in the location of the most isometric zone, its extent and exact position differ between specimens, underscoring the need for a personalized approach.

To enhance the clinical relevance of the model and assess the surgical feasibility of candidate implant sites for the femoral bone tunnel entry sites, we iteratively calculated the diameter of a zone within the medial femoral condyle based on different strain-based isometry thresholds. In all specimens, a 5 mm diameter region which included only points remaining below a 9% strain threshold was identified. Its centroid was located cranioproximally to that of the native CrCL footprint, consistently lying outside of it. The strain thresholds (6.5–9%) are consistent with human ACL near-isometric replacement data, where an allowable 1–3 mm (16, 19, 20) elongation relative to an average ligament length of approximately 30 mm (50) corresponds to a strain of about 3–10%.

Furthermore, depending on the tolerated strain rate, we found that not all the nodes within the CrCL footprint would be suitable for implantation. If an anatomical implantation at the native CrCL footprint were considered, this would need to be tailored to the animal and the intrinsic properties of the SL, and may not guarantee to meet the threshold over the entire insertion surface. These findings also suggest that, in certain cases, using of smaller SL that require reduced tunnel diameters may be preferable to better accommodate individual anatomical variability.

These results further underscore the importance of patient-specific biomechanical modeling for selection of a SL with appropriate yield strength and optimization of its positioning before considering intra-articular implantation in dogs. Our findings may help to guide patient-specific implantation, based on selecting the most suitable SL according to its biomechanical properties and the required surface area for anchoring.

Some limitations of the study must be acknowledged. The small number of specimens prevents statistical inference. The inclusion of dogs from different large-breed populations predisposed to CrCL rupture intentionally broadened the anatomical diversity of the dataset. These animals exhibited a wide range of sizes and morphologies, even among animals of similar weights.

More importantly, the isometry assessment relied on a conservative worst-case approach, as described in the Methods section. This approach may limit the extent of the predicted isometric zones; however, when considering the 3D architecture of a SL as a whole, the functional isometric regions may be more permissive than predicted by this point-based assessment.

Another limitation lies in the geometrical assessment of isometry with interinsertion distance variations based on a simplified representation of the intra-articular trajectories, modeled as either a single straight segment between two points or as a bundle of independent line elements between paired points. This approach is consistent with most ACL and CrCL studies, in which isometry is usually assessed by measuring length variations between pairs of femorotibial points, with the ligament modeled as a line connecting these two points (17, 46, 47). Such modeling allowed us to assess trajectories length variations and ensure axial stress remains below experimentally determined failure thresholds of the most commonly used SLs (43). However, it does not account for SL stiffness, nonlinear structural behavior, or the influence of 3D architecture. In braided or helical designs, axial strain is mechanically coupled with fiber reorientation and torsional effects (51). These interactions may generate internal shear stresses and stress concentrations, particularly near fixation sites, potentially leading to fatigue damage even when axial elongation remains below ultimate limits. Consequently, trajectories that appear geometrically isometric within a purely kinematic 3D model may not be mechanically isometric once replaced by a structurally complex SL. The predicted strain should therefore be interpreted as simplified axial elongation rather than a direct representation of the SL’s functional mechanical environment *in vivo*, underscoring the need for future models incorporating torsional and shear-related effects and SL architecture.

Last but not least, variations in distance during the flexion–extension cycle were calculated in stifles with intact CrCLs under unloaded conditions. While these results provide valuable insights into isometry, it should be noted that these conditions may not accurately reflect functional isometry or the deformation of trajectories that occur under physiological load. Specifically, they do not take into account the stabilizing role of ligaments and muscles in the stifle joint (52), nor the change in kinematics following CrCL failure (53, 54) which may be further exacerbated in dogs with increased tibial slope.

One way to overcome this limitation would be to study the effect of different implantation sites on stifle kinematics using a musculoskeletal model. Currently, no studies in the literature consider isometry and variations in kinematics due to changes in implantation sites. A model that considers both muscle effort and kinematics would enable the computer model to be used in a clinical setting.

Beyond isometry, impingement with other intra-articular structures must also be considered, as contact-induced wear and release of debris, provoke synovitis, and accelerate SL degradation. Further studies are needed to assess the optimal implantation sites, with this parameter also being considered.

Despite its limitations, this study identified a zone suitable for standard 5 mm tunnel drilling across all specimens. This zone was located cranio-proximally to the centroid of the native CrCL footprint, with associated strain thresholds (6.5–9%) consistent with human ACL near-isometric replacement data. This suggests that such positioning could minimize SL overstrain and excessive stress at the fixation interface.

Future developments should incorporate additional determinants of failure, including physiological loading conditions involving coupled torsional and shear stresses, as well as the impact of individual tibial slope, SL geometry and its intraoperative tensioning. They should also consider SL-bone interactions, including the potential for mechanical impingement or collision with surrounding bony structures, as well as the SL-to-bone fixation mode, encompassing tunnel angulation and the type of anchorage devices used.

Beyond its experimental application, the methodology developed in this study may represent a first step toward the development of a patient-specific digital twin integrated into the surgical workflow for intra-articular SL reconstruction. A logical next step would be to evaluate this approach *in vivo* by combining a preoperative CT scan with dynamic single-plane fluoroscopy of passive, unloaded stifle flexion–extension under general anaesthesia, reproducing the intra-operative manoeuvres routinely used during human ACL reconstruction to assess graft isometry. Model-based 2D-3D image registration could then reconstruct subject-specific stifle kinematics, providing a further step toward patient-specific identification of isometric insertion sites and optimization of implant positioning.

## 5 Conclusion

This study demonstrated the feasibility of using a subject-specific 3D modeling approach to optimize intra-articular CrCL replacement in dogs. The proposed CT-based method, combined with ex vivo-measured kinematics, enabled precise animal-specific mapping of femoral sites for the placement of SLs within intra-articular trajectories which limit strain during flexion–extension. This novel methodological framework paves the way for personalized, image-guided surgical planning. The ultimate goal is to overcome the current limitations of SLs implantation and improve long-term joint stability with the aim of transferring this to both human and veterinary clinical settings. However, future refinement of the 3D kinematic model integrating active loading, soft-tissue mechanics, and SL architecture is required to support clinical translation.

## Data Availability

The raw data supporting the conclusions of this article will be made available by the authors, without undue reservation.
